# Demonstration of reconfigurable joint orbital angular momentum mode and space switching

**DOI:** 10.1038/srep37331

**Published:** 2016-11-21

**Authors:** Jun Liu, Jian Wang

**Affiliations:** 1 Wuhan National Laboratory for Optoelectronics, School of Optical and Electronic Information, Huazhong University of Science and Technology, Wuhan 430074, Hubei, China

## Abstract

We propose and demonstrate space-selective switch functions employing orbital angular momentum (OAM) modes in the space domain for switching network. One is the switching among different OAM modes having different spatial phase structures, called OAM mode switching. The other is the switching among different space locations, called space switching. The switching operation mechanism relies on linear optics. Reconfigurable 4 × 4 OAM mode switching, space switching, and joint OAM mode and space switching fabric using a single spatial light modulator (SLM) are all demonstrated in the experiment. In addition, the presented OAM-incorporated space-selective switch might be further extended to N × N joint OAM mode and space switching with fast response, scalability, cascading ability and compability to facilitate robust switching applications.

Unfettered data switching occurs between individuals, businesses and the cloud at any moment over the world by utilizing pervasive information access available to us in various forms (smart phones, laptops, and wearable devices such as watches and glasses)^1^. Optical networks are the interconnection fabric of the global internet, including undersea links across the oceans, terrestrial networks connecting continents, countries, and cities, and ending with points of business and home^2^. However, the unabated exponential growth of global internet traffic is driving an ever-increasing demand for higher data capacity and more efficient spectral usage in transmission links. To sustain the exploding capacity demands, wavelength-division multiplexing (WDM) allowing multiple frequency-separated channels to co-transmit is adapted^3^,^4^,^5^. In addition, using modulation formats that encode information over amplitude, phase, and polarization at each wavelength channel can further increase the capacity of optical transmission links^6^,^7^,^8^,^9^. Modern optical networks are formed by nodes that transmit, receive and route data, which are interconnected by links. Mesh network nodes are linked to three or four neighbouring nodes with each link carrying two-way traffic typically. Transparent switching at each node of network links, or cross connect functionality, is required for realizing an all-optical network^10^. The wavelength-selective switch (WSS) fulfils all the mesh networking requirements above for the WDM system^11^,^12^. Yet recently space-division multiplexing (SDM) exploiting the transverse spatial structure dimension of light beams has attracted more and more attention to meet the capacity requirements^13^. Few-mode fiber (FMF) and multi-core fiber (MCF) have gained great success in SDM for efficient increase of fiber optical transmission capacity^14^,^15^,^16^,^17^. Analogical to WDM system, we need a space-selective switch working as a “WSS” in SDM system^18^,^19^,^20^. Very recently, orbital angular momentum (OAM), which is also related to the spatial phase structure of an electromagnetic wave, has shown its possible applications both in free-space and fiber transmission links^21^,^22^,^23^,^24^,^25^,^26^,^27^,^28^,^29^. Remarkably, similar to other mode bases in free space or fiber, OAM modes are another basis with which to represent spatial modes. We could use different mode bases for SDM, and so does OAM modes. An OAM beam features a spiral phase front of 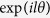 in which 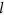 is the topological charge value and 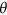 refers to the azimuthal angle. The distinct features are unlimited charge values of OAM and intrinsic orthogonality among different OAM states which facilitate an alternative multiplexing technique, i.e. OAM-division multiplexing. In this scenario, it would be valuable to develop OAM-incorporated space-selective switch when exploiting OAM modes in SDM related applications.

In this paper, we present two proof-of-concept space-selective switch functions employing OAM modes in the space domain for switching network. One is the switching among different OAM modes having different spatial phase structures, which is called OAM mode switching. The other is the switching among different space locations, which is called space switching. Moreover, we also report a reconfigurable joint OAM mode switch and space switch fabric using a single spatial light modulator (SLM). 4 × 4 OAM mode switching, space switching and joint OAM mode and space switching are all demonstrated in the experiment.

## Results

### Concept of OAM mode switching, space switching and joint OAM mode and space switching

**Figure 1:**
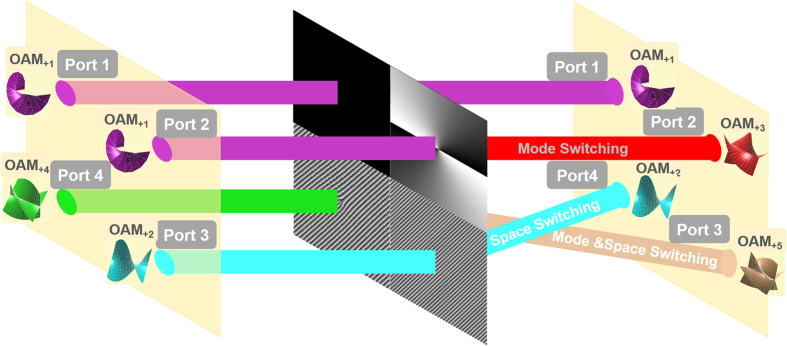
Concept of OAM mode switching, space switching, and joint OAM mode and space switching.

Figure 1 illustrates the concept of OAM mode switching, space switching, and joint OAM mode and space switching. One can see four typical switching examples. Case 1: the same OAM mode (e.g. OAM_+1_) is delivered straightforward from input port 1 to the same output port 1, i.e. without OAM mode switching and without space switching. Case 2: one OAM mode (e.g. OAM_+1_) at input port 2 is switched to another OAM mode (e.g. OAM_+3_) at the same output port 2, i.e. with OAM mode switching and without space switching. Case 3: The same OAM mode (e.g. OAM_+2_) is switched from input port 3 to the different output port 4, i.e. without OAM mode switching and with space switching. Case 4: one OAM mode (e.g. OAM_+4_) at input port 4 is switched to another OAM mode (e.g. OAM_+5_) at the different output port 3, i.e. with OAM mode switching and with space switching (joint OAM mode and space switching). For simplicity we call Case 1 no switching, Case 2 OAM mode switching, Case 3 space switching, and Case 4 joint OAM mode and space switching.

### Experimental setup of reconfigurable 4 × 4 OAM mode switching, space switching and joint OAM mode and space switching

**Figure 2:**
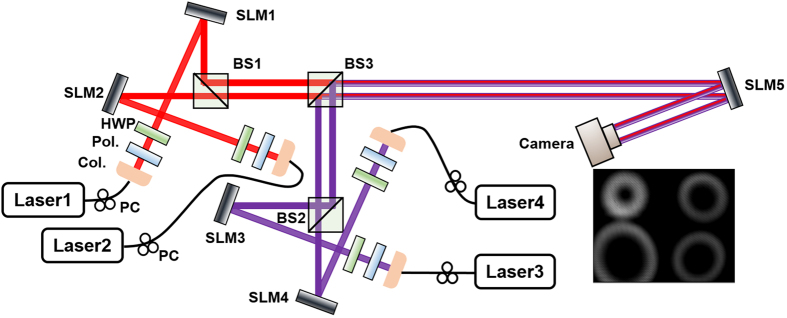
Experimental setup of reconfigurable 4 × 4 OAM mode switching, space switching, and joint OAM mode and space switching. PC: polarization controller. Col.: collimator. Pol.: polarizer. HWP: half-wave plate. BS1–3: non-polarizing beam splitter. SLM1-5: spatial light modulator.

The proof-of-concept experimental setup of reconfigurable 4 × 4 OAM mode switching, space switching, and joint OAM mode and space switching is illustrated in Fig. 2. Four lasers and four SLMs are employed to prepare four channels located at four different spatial positions having different OAM states. Each channel delivers a particular OAM mode to a specific location in the space domain. SLM1-SLM4 are employed to generate four different OAM modes which are then gathered by three non-polarizing beam splitters (BS1-BS3). The four light beams carrying different OAM modes propagate in parallel after the three BSs. The BS1 gathers light beams from SLM1 and SLM2 branches at the same height but separated horizontally. The BS2 gathers light beams from SLM3 and SLM4 branches at another height level while separated horizontally. The topological charge values of OAM modes generated from SLM1, SLM2, SLM3 and SLM4 branches are 1, 2, 3 and 4, respectively. SLM5 emulates the 4 × 4 switching node. The four input ports of the switching node are linked to the four OAM channels after BS3 and the four output ports are projected to a camera for monitoring. The inset of Fig. 2 depicts a typical intensity profile after BS3 with four different OAM modes located at four different spatial positions.

### Reconfigurable 4 × 4 OAM mode switching, space switching, and joint OAM mode and space switching

**Figure 3:**
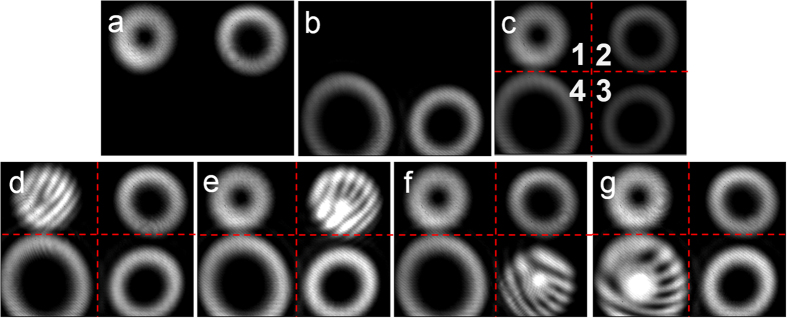
Different OAM modes generated by the four SLMs. (**a**) Observed intensity profile after BS1 generated by SLM1 and SLM2 with 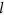 = 1 and 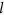 = 2, respectively. (**b**) Observed intensity profile after BS2 generated by SLM3 and SLM4 with 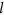 = 3 and 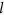 = 4, respectively. (**c**) Observed intensity profile after BS3. (**d**–**g**) Measured interference patterns between a reference Gaussian beam and OAM modes (**d**) 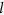 = 1, (**e**) 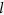 = 2, (**f**) 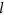 = 3, (**g**) 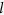 = 4 at input ports.

Figure 3(a) shows the observed intensity profile after BS1 which is generated by SLM1 and SLM2, and the topological charge value 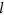 is 1 and 2, respectively. Figure 3(b) shows the observed intensity profile after BS2 which is generated by SLM3 and SLM4 with 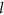 = 3 and 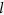 = 4. The horizontally separated light beams from SLM3 and SLM4 are at the same height level but different from those light beams from SLM1 and SLM2. The four light beams from SLM1-SLM4 are gathered together by BS3 to form a rectangular shape before linking to the switching node (SLM5), as shown in Fig. 3(c). The four spatial positions of four light beams from SLM1-SLM4 before SLM5 correspond to the four input ports 1 to 4 of the switching node. The four output ports of the switching node correspond to the four spatial positions of four light beams after the SLM5 which is captured by the camera. The switching function is achieved by SLM5 which is divided into four parts to independently steer the four light beams from four input ports and deliver them to four output ports. Figure 3(d–f) show measured interference patterns (i.e. interferograms) between a reference Gaussian beam and the OAM modes with a slight tilted angle at the input ports. Note that the number and direction of forks in the interferograms imply the magnitude and sign of the topological charge value. One can clearly see that the topological charge value of OAM mode at input port 1, port 2, port 3 and port 4 is 1, 2, 3 and 4, corresponding to SLM1 to SLM4 branches, respectively.

**Figure 4:**
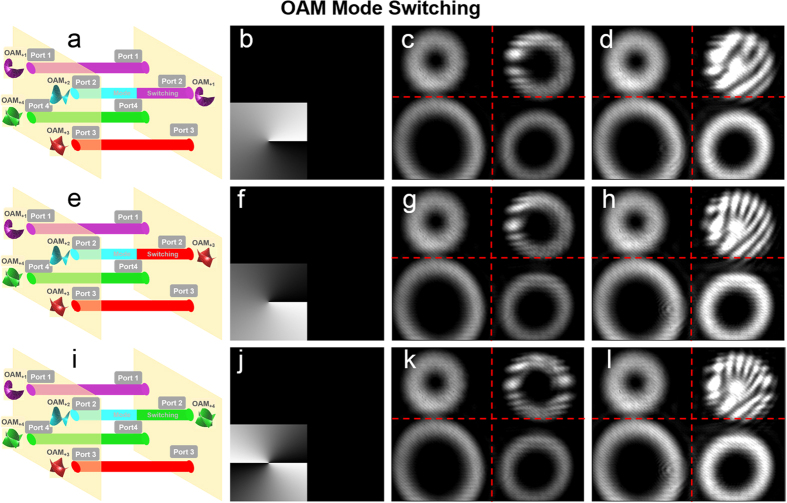
(**a**,**e**,**i**) OAM mode switching in port 2. (**b**,**f**,**j**) Phase patterns loaded to SLM5 to realize OAM mode switching. (**c**,**g**,**k**) Intensity profiles after OAM mode switching in port 2. (**d**,**h**,**l**) Interference patterns of the OAM modes with a reference a Gaussian beam after OAM mode switching.

We first demonstrate the process of OAM mode switching. As illustrated in Fig. 4(a,e,i), port 2 is taken as an example to show OAM mode switching from 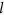 = 2 to 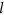 = 1, 3, 4. The phase patterns loaded to the SLM5 are shown in Fig. 4(b,f,j). The measured intensity profiles after OAM mode switching from 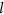 = 2 to 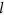 = 1, 3, 4 are shown in Fig. 4(c,g,k), respectively. Figure 4(d,h,l) depict the corresponding interference patterns of the OAM modes with a reference Gaussian beam after OAM mode switching. Remarkably, as the imaging process is upside down and laterally reversal, the location of phase pattern (bottom left) is reverse with respect to the intensity profile (top right). To clearly show the OAM mode switching function, one input port of four ports is chosen to perform the OAM mode switching.

**Figure 5:**
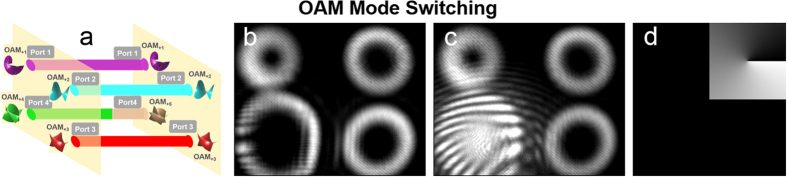
(**a**) OAM mode switching to a new topological charge value in port 4. (**b**) Phase pattern loaded to SLM5 to realize OAM mode switching. (**c**) Intensity profile after OAM mode switching in port 4. (**d**) Interference pattern of OAM mode with a reference Gaussian beam after OAM mode switching.

We also demonstrate the OAM mode switching, in which OAM mode is switched to a new topological charge value, e.g. 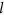 = 5, as illustrated in Fig. 5(a). The phase pattern loaded to the SLM5 is shown in Fig. 5(b). The measured intensity profile after OAM mode switching from 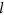 = 4 to 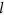 = 5 is shown in Fig. 5(c). Figure 5(d) depicts the interference patterns of the OAM mode with a reference Gaussian beam after OAM mode switching.

**Figure 6:**
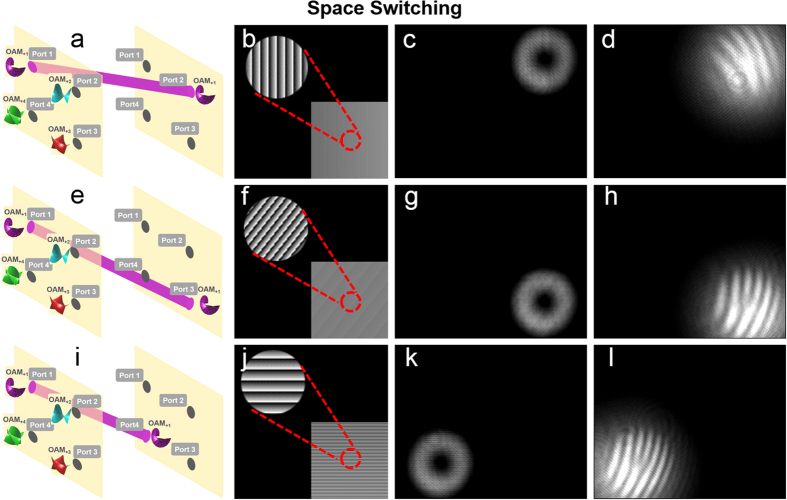
(**a**,**e**,**i**)Space switching from port 1 to port 2, port 3 and port 4. (**b**,**f**,**j**) Phase patterns loaded to SLM5 to realize space switching. (**c**,**g**,**k**) Intensity profiles after space switching in port 2, port 3 and port 4, respectively. (**d**,**h**,**l**) Interference patterns of the OAM modes with a reference Gaussian beam after space switching.

We then demonstrate the process of space switching. As illustrated in Fig. 6(a,e,i), port 1 is taken as an example to show space switching from input port 1 to output port 2, port 3 and port 4, respectively. The phase patterns loaded to the SLM5 are shown in Fig. 6(b,f,j). The insets of Fig. 6(b,f,j) depict enlarged phase patterns with more details. The intensity profiles after space switching are shown in Fig. 6(c,g,k), respectively. Figure 6(d,h,l) depict the corresponding interference patterns of the OAM modes with a reference Gaussian beam after space switching. The topological charge value 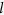 of OAM mode at output port 2, port 3, and port 4 is 1, implying the realization of space switching without OAM mode switching.

**Figure 7:**
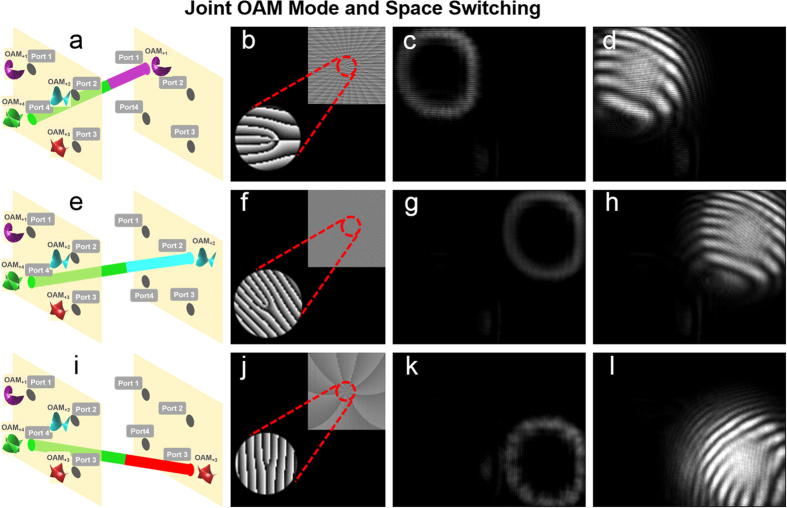
(**a**,**e**,**i**) Joint OAM mode and space switching. (**b**,**f**,**j**) Phase patterns loaded to SLM5 to realize joint OAM mode and space switching. (**c**,**g**,**k**) Intensity profiles after joint OAM mode and space switching in port 1, port 2 and port 3, respectively. (**d**,**h**,**l**) Interference patterns of the OAM modes with a reference Gaussian beam after joint OAM mode and space switching.

We further demonstrate the process of joint OAM mode and space switching. As illustrated in Fig. 7(a,e,i), port 4 is taken as an example to show joint OAM mode and space switching from OAM_+4_ at input port 4 to OAM_+1_ at output port 1, OAM_+2_ at output port 2, and OAM_+3_ at output port 3, respectively. The phase patterns loaded to the SLM5 are shown in Fig. 7(b,f,j). The insets of Fig. 7(b,f,j) depict enlarged phase patterns with more details. The measured intensity profiles after joint OAM mode and space switching are shown in Fig. 7(c,g,k), respectively. Figure 7(d,h,l) depict the corresponding interference patterns of the OAM modes with a reference Gaussian beam after joint OAM mode and space switching. The topological charge value 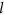 of OAM mode at output port 1, port 2 and port 3 is 1, 2, and 3, respectively, indicating the successful implementation of joint OAM mode and space switching.

**Figure 8:**
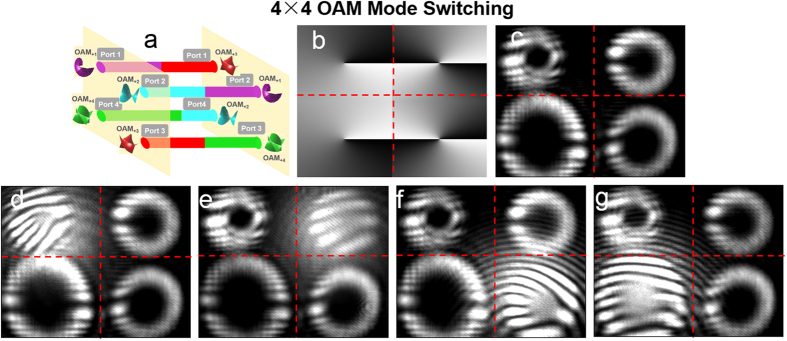
(**a**) 4 × 4 OAM mode switching. (**b**) Phase pattern loaded to SLM5 to realize 4 × 4 OAM mode switching. (**c**) Intensity profiles after 4 × 4 OAM mode switching. (**d**–**g**) Interference patterns of the OAM modes with a reference Gaussian beam after 4 × 4 OAM mode switching.

For clear show of different kinds of switching operations, the obtained results in Figs 4, 5, 6, 7 apply to only one input port. To further verify the robust switch operations, reconfigurable 4 × 4 OAM mode switching, space switching and joint OAM mode and space switching are also demonstrated in the experiment. Figure 8(a) illustrates the process of 4 × 4 OAM mode switching. The phase pattern loaded to the SLM5 is shown in Fig. 8(b). The intensity profiles after 4 × 4 OAM mode switching are shown in Fig. 8(c). Figure 8(d–g) depict the interference patterns of the OAM modes with a reference Gaussian beam after 4 × 4 OAM mode switching. The topological charge value 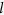 of OAM mode at output port 1, port 2, port 3 and port 4 is switched from 1 to 3, 2 to 1, 3 to 4 and 4 to 2, respectively.

**Figure 9:**
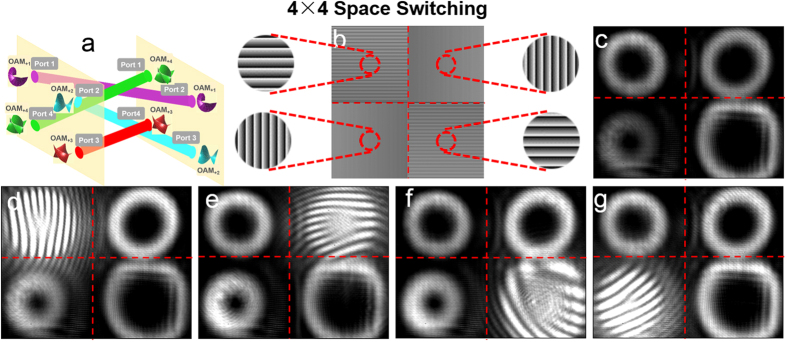
(**a**) 4 × 4 space switching. (**b**) Phase pattern loaded to SLM5 to realize 4 × 4 space switching. (**c**) Intensity profiles after 4 × 4 space switching. (**d**–**g**) Interference patterns of the OAM modes with a reference Gaussian beam after 4 × 4 space switching.

Figure 9(a) illustrates the process of 4 × 4 space switching. The obtained results of 4 × 4 space switching are shown in Fig. 9(b–g). The phase pattern loaded to the SLM5 is shown in Fig. 9(b). The insets of Fig. 9(b) depict enlarged phase patterns with more details. The intensity profiles after 4 × 4 space switching are shown in Fig. 9(c). Figure 9(d–g) depict the interference patterns of the OAM modes with a reference Gaussian beam after 4 × 4 space switching. OAM modes 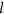 = 1, 2, 3, 4 at input port 1, port 2, port 3 and port 4 are spatially switched to output port 4, port 1, port 2 and port 3, respectively.

**Figure 10:**
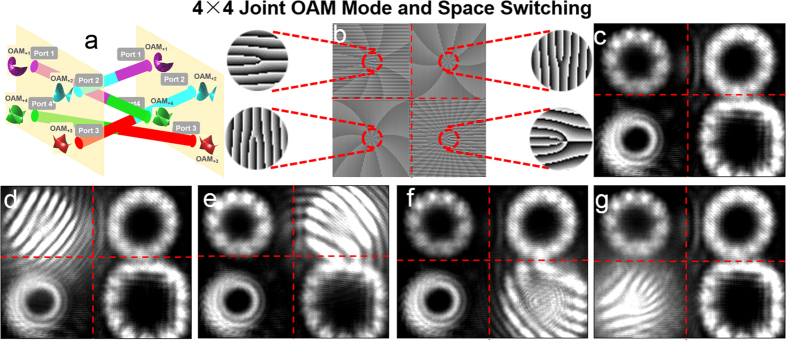
(**a**) 4 × 4 joint OAM mode and space switching. (**b**) Phase pattern loaded to SLM5 to realize 4 × 4 joint OAM mode and space switching. (**c**) Intensity profiles after 4 × 4 joint OAM mode and space switching. (**d**–**g**) Interference patterns of the OAM modes with a reference Gaussian beam after 4 × 4 joint OAM mode and space switching.

Figure 10(a) illustrates the process of 4 × 4 joint OAM mode and space switching. The obtained results of 4 × 4 joint OAM mode and space switching are shown in Fig. 10(b–g). The phase pattern loaded to the SLM5 is shown in Fig. 10(b). The intensity profiles after joint OAM mode and space switching are shown in Fig. 10(c). Figure 10(d–g) depict the interference patterns of the OAM modes with a reference Gaussian beam after joint OAM mode and space switching. OAM modes 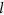 = 1, 2, 3, 4 at input port 1, port 2, port 3 and port 4 are spatially switched to output port 4, port 1, port 2 and port 3 together with updated OAM modes 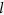 = 4, 1, 2, 3, indicating successful implementation of 4 × 4 joint OAM mode and space switching.

## Discussion

In summary, by exploiting linear optics operation mechanism, we propose and demonstrate several kinds of OAM-incorporated space-selective switch functions, i.e. OAM mode switching, space switching, and joint OAM mode and space switching. We experimentally demonstrate reconfigurable 4 × 4 OAM mode switching, space switching and joint OAM mode and space switching using a single SLM. With future improvement to 4 × 4 switching, N × N joint OAM mode and space switching with fast response, scalability, cascading ability and compatibility might be achieved to facilitate robust N × N space-selective switch function.

### Response time

It is noted that the switching time of SLM based on nematic liquid crystal on silicon could be a bottleneck for fast-switch of OAM channels. To alleviate this problem, several possible solutions could be considered: (1) exploring transient nematic effects and phase wrapping techniques^30^; (2) employing ferroelectric liquid crystal SLM^31^,^32^; (3) adapting digital micromirror device (DMD)^33^; (4) using fast spatial light modulation optoelectronic devices^34^.

### Scalability

**Figure 11:**
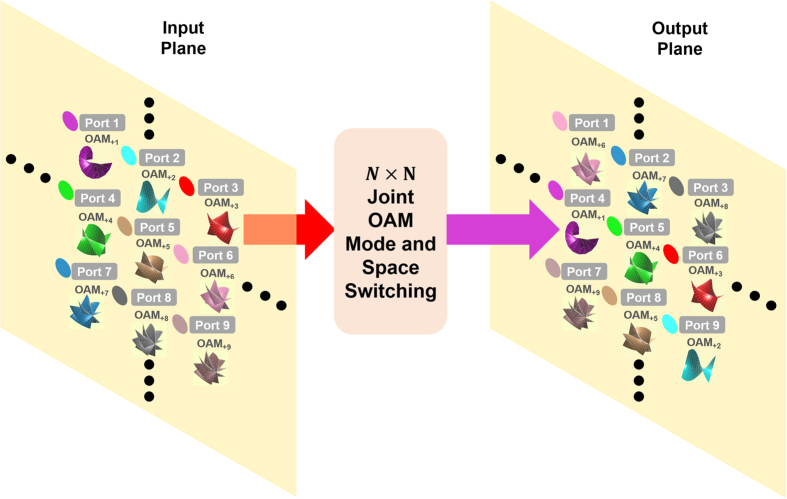
Concept of N × N joint OAM mode and space switching fabric.

The obtained results shown in Figs 4, 5, 6, 7, 8, 9, 10 show successful realization of reconfigurable 4 × 4 OAM mode switching, space switching, and joint OAM mode and space switching using a single SLM. The operation mechanism relies on linear optics and different kinds of switching functions are demonstrated in the experiment. It is noted that the proposed space-selective switch is scalable. With further improvement, space-selective switch might be extended to N × N switching based on similar linear optics operation mechanism. Figure 11 illustrates the concept of N × N joint OAM mode and space switching fabric. By employing a single SLM divided into N × N array with each unit independently loaded with a specific pattern, for the N × N OAM array with different OAM modes at different spatial locations, N × N OAM mode switching, space switching, or joint OAM mode and space switching from input plane to output plane could be achieved. However, the limited chip plane area of SLMs could limit the port number of NxN OAM mode and space switching^35^.

1. In the current experiment of 4 × 4 OAM mode and space switching, only part area of the SLM is utilized. Thus it is still possible to further extend the port number beyond 4 × 4 switching by fully utilizing the effective area of SLMs. The SLMs employed in the experiments are Holoeye PLUTO phase-only SLMs based on reflective liquid crystal on silicon (LCOS). These SLMs have a spatial resolution of 1920  ×  1080 pixels, a small pixel pitch size of 8 μm, and an active area of 15.36  ×  8.64 mm. The diameter of light beams is about 3 mm. Thus, the extended port number could be estimated to be ~10. Actually, the beam size can be reduced using lens pair to further increase the port number of OAM and space switching.
2. To increase the port number, on one hand, SLM with relatively larger chip plane area could be employed; on the other hand, multiple SLMs could be combined together but with relatively increased system complexity.
3. To improve the switch channel density, OAM modes with relatively lower-order topological charge values or even fractional topological charge values could be considered^36^,^37^. Since lower-order OAM modes have relatively smaller beam sizes, it is possible to increase the switch channel density not only with small OAM mode spacing but also with small space distance.

### Cascading ability

**Figure 12:**
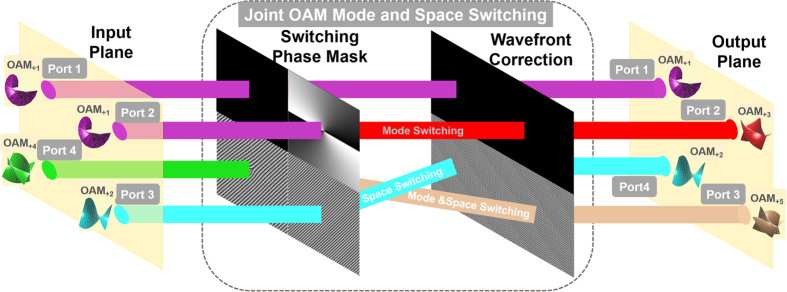
Concept of joint OAM mode and space switching with multiple light beams in parallel both in input plane and output plane. An additional wavefront correction stage is added.

Remarkably, as shown in Fig. 1, the multiple light beams at the input ports propagate in parallel while become crossed with each other at the output ports after switching. This could cause some problems when collecting the multiple light beams after switching to other communication systems. Also, it could cause problems when cascading multiple switching unites. So the ideal case after switching would be to also have multiple light beams at the output ports propagate in parallel. As shown in Fig. 12, this might be achieved by adding an additional wavefront correction stage, which could be simple grating phase pattern. By employing the switching unit as shown in Fig. 12, it is expected to improve the N × N OAM mode switching, space switching, and joint OAM mode and space switching with potential cascading ability.

### Compatibility

Additionally, it is desirable that the presented OAM mode and space switching could be also compatible with the existing single mode fiber (SMF) based optical network. In this scenario, one could combine inverted spiral phase pattern with the grating phase pattern in the wavefront correction state shown in Fig. 12. As a consequence, the OAM modes after mode and space switching could be back converted to Gaussian-like beams with bright spot at the beam center which can be easily coupled into SMF.

## Methods

**Figure 13:**
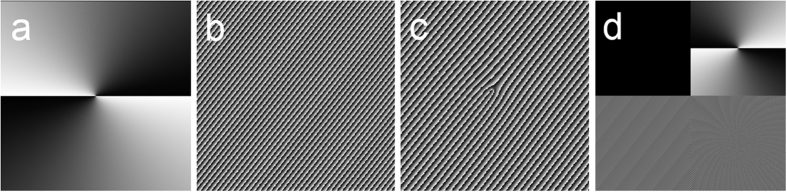
(**a**) Phase pattern used to achieve mode switching. (**b**) Phase pattern used to achieve space switching. (**c**) Phase pattern used to achieve joint OAM mode and space switching. (**d**) Phase pattern with four parts loaded to a single SLM to achieve 4 × 4 OAM mode switching, space switching, and joint OAM mode and space switching.

Figure 13(a) shows a typical spiral phase pattern to change the topological charge value of an OAM mode to achieve OAM mode switching function. A typical phase pattern to enable space switching is shown in Fig. 13(b). It could be a grating phase pattern with different direction and period to steer the OAM light beam to a different location in the space domain. The combination of the spiral phase pattern and grating phase is displayed in Fig. 13(c), which could be used to achieve the joint OAM mode and space switching. Figure 13(d) shows the phase pattern loaded to a single SLM to perform 4 × 4 OAM mode switching, space switching, and joint OAM mode and space switching. The pattern is divided into four parts and each part is loaded with a different pattern discussed above according to different switching functions. As a consequence, one can independently steer four input light beams for individual OAM mode switching, space switching or joint OAM mode and space switching. Moreover, reconfigurable 4 × 4 OAM mode switching, space switching, and joint OAM mode and space switching are also available simply by changing the pattern loaded to the single switching SLM (i.e. SLM5 in Fig. 2).

## Additional Information

**How to cite this article**: Liu, J. and Wang, J. Demonstration of reconfigurable joint orbital angular momentum mode and space switching. *Sci. Rep.* **6**, 37331; doi: 10.1038/srep37331 (2016).

**Publisher’s note:** Springer Nature remains neutral with regard to jurisdictional claims in published maps and institutional affiliations.
